# Coping Under Pressure : Psychoactive Substance Use Among Psychiatric Nurses in Tunisia

**DOI:** 10.1192/j.eurpsy.2026.10896

**Published:** 2026-07-31

**Authors:** M. Bouchendira, A. Maamri, A. Amara, A. Karmous, A. Hajri, H. Zalila

**Affiliations:** 1 Emergencies and consultations, RAZI hospital, Manouba; 2 Community and Preventive Medicine, Faculty of medicine of Sousse, Sousse, Tunisia

## Abstract

**Introduction:**

Psychiatric nurses face high occupational stress, frequently exposed to patient aggression and emotionally charged situations. These pressures may be associated with maladaptive coping strategies, including psychoactive substance use. Investigating these patterns can inform preventive interventions and support mental well-being.

**Objectives:**

To assess the prevalence of psychoactive substance use among psychiatric nurses in Tunisia and examine its association with coping strategies and psychological distress.

**Methods:**

An exhaustive cross-sectional study including 237 psychiatric nurses was conducted from January 2024 to April 2024. Participants completed a self-administered questionnaire assessing the use of tobacco, alcohol, and cannabis , coping strategies via the Brief COPE inventory and psychological distress using the DASS-21 scale.

**Results:**

Substance use was reported by 21.9% (tobacco), 7.6% (alcohol), and 1.7% (cannabis). The most common coping strategies were religion (51.9%), planning (49.4%), and acceptance (47.3%). Avoidant coping strategies including denial, behavioral disengagement, and substance use ,were significantly associated with higher psychological distress (p < 0.05).

Younger nurses (<35 years) relied more on avoidant coping (38% vs. 21%) and reported higher substance use (tobacco 28% vs. 18%, alcohol 11% vs. 5%). Nurses with fewer than 10 years of experience also showed higher avoidant coping (36% vs. 20%) and substance use (tobacco 26% vs. 18%, alcohol 10% vs. 5%), identifying vulnerable subgroups for targeted interventions.

This study did not assess psychotropic use or addiction, which may be relevant due to their accessibility in psychiatric settings.

**Conclusions:**

Most psychiatric nurses employ adaptive coping strategies, yet a minority use psychoactive substances linked to avoidant coping and higher psychological distress. Younger and less experienced nurses are particularly at risk. Findings support institutional measures, adaptive coping training, and early identification of at-risk staff. Future longitudinal studies are needed to explore these associations further.

**Disclosure of Interest:**

None Declared

